# The know-do gap in sick child care in Ethiopia

**DOI:** 10.1371/journal.pone.0208898

**Published:** 2018-12-12

**Authors:** Anna D. Gage, Margaret E. Kruk, Tsinuel Girma, Ephrem T. Lemango

**Affiliations:** 1 Department of Global Health and Population, Harvard T.H. Chan School of Public Health, Boston MA, United States of America; 2 Department of Global Health and Population, Harvard T.H. Chan School of Public Health, Addis Ababa, Ethiopia; 3 International Institue for Primary Health Care- Ethiopia, Addis Ababa, Ethiopia; New York City Department of Health and Mental Hygiene, UNITED STATES

## Abstract

**Background:**

While health care provider knowledge is a commonly used measure for process quality of care, evidence demonstrates that providers don’t always perform as much as they know. We describe this know-do gap for malaria care for sick children among providers in Ethiopia and examine what may predict this gap.

**Methods:**

We use a 2014 nationally-representative survey of Ethiopian providers that includes clinical knowledge vignettes of malaria care and observations of care provided to children in facilities. We compare knowledge and performance of assessment, treatment and counseling items and overall. We subtract performance scores from knowledge and use regression analysis to examine what facility and provider characteristics predict the gap. 512 providers that completed the malaria vignette and were observed providing care to sick children were included in the analysis.

**Results:**

Vignette and observed performance were both low, with providers on average scoring 39% and 34% respectively. The know-do gap for assessment was only 1%, while the gap for treatment and counseling items was 39%. Doctors had the largest gap between knowledge and performance. Only provider type and availability of key equipment significantly predicted the know-do gap.

**Conclusions:**

While both provider knowledge and performance in sick child care are poor, there is a gap between knowledge and performance particularly with regard to treatment and counseling. Interventions to improve quality of care must address not only deficiencies in provider knowledge, but also the gap between knowledge and action.

## Introduction

The child mortality rate in Ethiopia has dropped substantially in the past twenty years, from 161 deaths per 1000 births in 1997 to just 58 in 2017 [1]. However, more progress is required in order to reach the Sustainable Development Goal target of 25 by 2030 [2]. Most children in Ethiopia die of causes that are preventable in the health system such as pneumonia and diarrhea [3]. To prevent these deaths, the health system has to provide competent evidence based-care, including thorough assessment, accurate diagnosis, appropriate treatment and clear counseling.

Evidence suggests that not all of these elements are being done and the quality of care provided to sick children is poor in Ethiopia and other sub-Saharan African countries [4–7]. The quality that health workers provide is inconsistent, varying from one patient to the next [8]. The poor and inconsistent quality could be a reflection of the know-do gap, or the difference between a provider’s knowledge of appropriate care and what they actually perform in a clinical visit. This gap has been documented among health care workers urban and rural settings in India and Tanzania [9–13].

Understanding this gap is critical to diagnosing causes of poor quality and informing improvement efforts. Clinical decision making is a complex process influenced by many factors in addition to the knowledge base. Many improvement strategies have focused exclusively on building knowledge in order to improve performance: an analysis of GAVI, Global Fund and World Bank support of human resources for health found that short-term in-service training was by far the most commonly funded activity [14]. However, such training will not improve health worker performance if it does not address the know-do gap. Indeed, in-service training is associated with only minor improvements in sick child management [15, 16].

In this paper, we describe the gap between knowledge and performance for sick child care in Ethiopia and examine the facility and provider factors which may contribute to this gap.

## Methods

We used cross-sectional facility data from the nationally representative 2014 Ethiopia Service Provision Assessment Plus (SPA+) survey [17]. The survey gathered data on facility and provider characteristics, provider knowledge and observations of clinical visits, including sick child visits. Because unique provider IDs were not available to directly link the provider knowledge tests with the observations of care, the analytic sample was drawn from providers who had been observed providing sick child care and had a knowledge test with the same facility identifier, cadre and sex. Observations that had non-unique matches on these variables were excluded from analysis.

We constructed a measure of the know-do gap of sick child malaria care using data from the provider knowledge tests and observations of sick child visits. Knowledge and performance scores were created from all items that were available in both the knowledge test and the observations from the Integrated Management of Childhood Illness (IMCI) guidelines [18]. The IMCI guidelines were chosen as an internationally recognized protocol, however the items included in the knowledge test and observations can be seen as elements of good medical practice, such as asking about symptoms and taking the child’s temperature. These elements should be covered in any health worker education program, regardless of specific training on IMCI. The provider knowledge score is based on the vignette on child malaria and anemia in the knowledge test (Table A in S1 Table). The interviewers describe a four-year old boy presenting with fever that has been worsening over time, and asks the provider what elements of history, examinations and tests the provider would perform, what their preliminary diagnosis is and the treatments and counseling the provider would give. The knowledge score is defined as the percent of assessment, treatment and counseling items correctly identified by the provider.

The provider performance score is the average percent of items observed being performed during all the sick child visits that the provider conducted. Testing for malaria was only defined for visits where the caregiver reported fever and prescribing artesunate or quinine was only defined for visits where the provider’s diagnosis was malaria. To more closely align the clinical observations with the vignette, we also calculated the entire performance score including only sick child visits where the caregiver reported child fever. We subtracted the performance score from the knowledge score to calculate the know-do gap score for each provider. As a sensitivity analysis, we alternately directly compared the similar the performance and knowledge indicators and calculated the know-do gap as the total number of discordant items.

We considered a range of facility environment and provider covariates that might predict the size of the know-do gap adapted from Rowe et al [19]. Facility environment variables included urban/rural, the ratio of sick child patients to providers on the day of the assessment, the facility’s basic amenities defined by the service readiness index (including electricity, water, privacy, sanitation, communication tools, computer and emergency transportation), specific tools necessary for assessing or treating the child (including a scale, thermometer, malaria diagnostics and anti-malarial drugs), and facility management. Facility management is defined as an index ranging from 0–1 of the presence of routine quality assurance procedures, a client feedback system, a health management information system, management and community meetings, and evidence of decisions and follow up from the management meetings. Provider characteristics considered include provider cadre, experience, motivation, any in-service training on care for sick children, and supervision in the past six months. Three aspects of provider motivation were considered: whether they receive a regular salary supplement, they have a written job description of their current position, and there are opportunities for promotion.

We present descriptive statistics of the sample facilities and providers and the know-do gap. We plotted the relationship between knowledge and performance. We examined the bivariate relationship between the know-do gap and our hypothesized predictors, using OLS regression and clustering at the facility level. All predictors that were significant at alpha = 0.2 were included in a multivariable OLS regression model to predict the know-do gap. All descriptive statistics are weighted, the regression is unweighted.

Analyses were conducted in Stata 14.1. The Harvard University Human Research Protection Program determined that this secondary analysis was exempt from review.

## Results

6,254 providers were interviewed as part of the SPA+ survey, of whom 3,947 ever provide sick child services. Of those providing sick child care, 590 providers completed both a provider test and were observed providing sick child care. 72 providers were excluded because they were not unique matches based on facility, provider sex and cadre. A further nine providers were excluded because the cadre was unknown, leaving 503 providers in the analytic sample. Table 1 presents characteristics of the sample. Nearly 50% of the sample was urban, and most worked in health centers. The health centers lacked many basic amenities, but most had key equipment for assessing and managing malaria, including a thermometer, scale and diagnostic tests. Nurses provided the majority of the sick child care. 426 of the providers saw at least one child whose caregiver reported a fever. The analytic sample is broadly similar to the full sample of providers who provide sick child care (Table B in S1 Table). The knowledge tests were not given in health posts nor given to health extension workers, leading to their exclusion in the analytic sample.

**Table 1:** Characteristics of sick child providers, N = 503.

	N	percent
Facility characteristics		
Urban	236	47%
Basic amenities (mean/sd)	0.43	0.22
Functional thermometer and scale	451	90%
Malaria diagnostics	366	73%
Sick child patients per provider	752	2.54
Management index	0.7	0.3
Facility type		
Hospital	54	11%
Health center	347	69%
Higher clinic	10	2%
Medium clinic	33	7%
Lower clinic	59	12%
Provider characteristics		
Female	180	36%
Years since graduation (mean/sd)	4.66	4.36
In-service training on sick children	225	45%
Recent supervision	307	61%
Receives regular salary supplement	408	81%
Has written job description	119	24%
Aware of opportunities for promotion	214	43%
Provider type		
MD	39	8%
Health officer	108	21%
Nurse/Midwife	356	71%

Table 2 displays the knowledge and performance scores for each IMCI assessment, treatment and counseling item and the gap between knowledge and performance. The most commonly known items were to take the child’s temperature (88%), do a malaria test (85%) and examine the child’s skin (65%), while the least commonly known item was to check for oedema (8%). The most commonly done items were to take a temperature (74%), ask about diarrhea (68%) and ask about cough (63%), while the least commonly done item was to count the child’s respirations (2%). There was a small gap between the knowledge and performance of assessment items (1%), but there was a much larger gap on treatment and counseling items (36%). For several items, particularly on asking about the child’s symptoms, providers scored better on performance than on knowledge. On average, providers scored 39% on knowledge and 34% on performance, resulting in a 6% overall know-do gap. Providers did not perform any better on assessment or counseling items when limited to visits where the caregiver reported a fever (Table C in S1 Table).

**Table 2:** Know-do gap for malaria care.

	Vignette average	Observed average	Gap
Assessment items			
Ask about seizures	22%	8%	14%
Ask about vomiting	36%	43%	-7%
Ask about eating during illness	36%	23%	14%
Ask about cough	59%	63%	-4%
Ask about diarrhea	36%	68%	-32%
Ask about vaccinations	11%	42%	-30%
Take temperature	88%	74%	14%
Weigh child	43%	46%	-3%
Check pallor	15%	27%	-12%
Check for oedema	8%	5%	3%
Examine skin	65%	19%	46%
Count respirations	12%	2%	10%
Examine neck	17%	20%	-3%
Examine mouth	17%	17%	0%
Do a malaria test^a^	85%	15%	70%
Assessment average	37%	36%	1%
Treatment and counseling items			
Prescribe artesunate or quinine^b^	59%	30%	29%
Counsel danger signs requiring return	41%	13%	28%
Counsel adherence to medication	58%	7%	50%
Treatment and counseling average	53%	17%	36%
Overall average	39%	34%	6%

^a^Only among febrile children in observed

^b^Only among children who have been diagnosed with malaria in observed

The knowledge and performance scores are displayed by provider cadre in Table 3. MDs scored higher than health officers and nurses on assessment knowledge, but scored the same as health officers and nurses on treatment and counseling knowledge and assessment performance. Nurses scored slightly better than MDs and health officers on treatment and counseling performance, however, their performance was still poor (0.18). All providers scored higher on knowledge than performance for treatment and counseling items; only MDs had a know-do gap on assessment items. The largest gap between knowledge and performance was in treatment and counseling among MDs.

**Table 3:** Know-do gap by domain and provider type.

	Vignette score	Observed score	Gap	T-test p value^1^
Assessment				
MD	50%	36%	14%	0.00
Health Officer	41%	37%	5%	0.23
Nurse	34%	36%	-2%	0.82
Treatment and counseling				
MD	54%	12%	42%	0.00
Health Officer	53%	14%	39%	0.00
Nurse	53%	18%	34%	0.00
Overall				
MD	51%	33%	18%	0.00
Health Officer	43%	34%	9%	0.52
Nurse	37%	34%	3%	0.00

^1^T test vignette = observed

**Fig 1:**
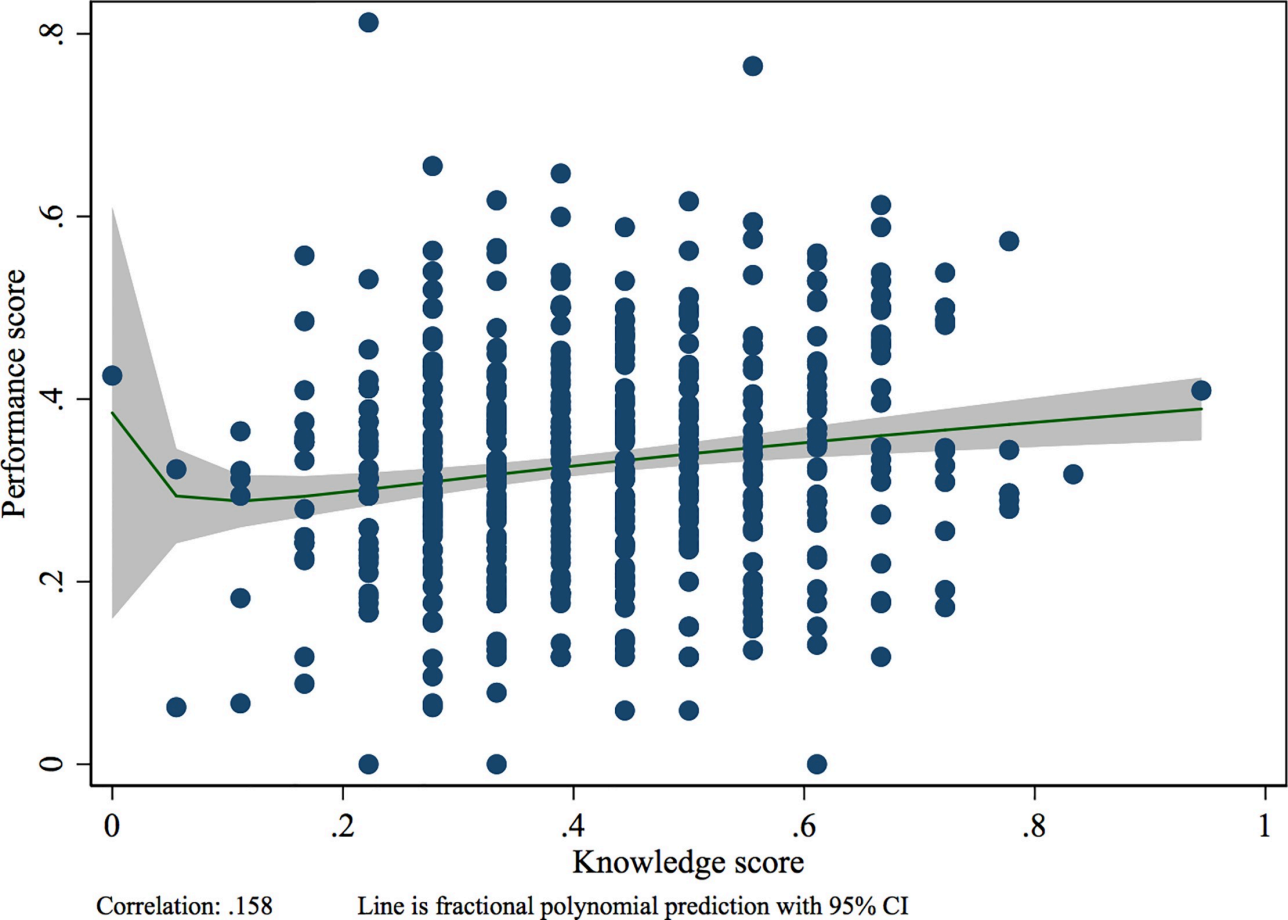
Relationship between vignette and observed performance among 503 providers.

Fig 1 plots the overall knowledge and performance scores with the fractional polynomial prediction line. While there is a slight positive correlation between knowledge and performance, there is enormous heterogeneity in performance among providers with similar knowledge scores. Notably, many providers score higher on performance than on knowledge. Controlling for the same covariates presented in Table 4, a 100% increase in knowledge is associated with a 15% increase in performance (Table D in S1 Table). Table 4 presents the results from the multivariable regression to predict the know-do gap. Of the covariates tested, only having a thermometer and the provider cadre were significantly associated with the know-do gap. Thermometer and scale availability was associated with a smaller gap, while doctors had a larger gap than nurses. These results were robust when calculating the gap only among observations of visits with febrile children and when alternately calculating the gap as the sum of the discordant performance and knowledge items (Table E in S1 Table).

**Table 4:** Multivariate predictors of know-do gap (N = 503).

	Beta	95% CI
Urban	0.03	(-.095,.07)
Basic amenities	0.06	(-.023,.142)
Thermometer and scale	-.063*	(-.125,-.001)
Malaria diagnostic	0.013	(-.026,.052)
In-service training on sick children	-0.026	(-.057,.005)
Recent supervision	-0.011	(-.045,.022)
Regular salary supplement	0.026	(-.016,.068)
Provider type		
MD	.09*	(.037,.143)
Health Officer	0.005	(-.032,.041)
Nurse/Midwife	(Reference)	
Constant	0.059	(-.017,.134)

* p < .05.

Basic amenities includes electricity, water, privacy, sanitation, communication tools, computer and emergency transportation. Recent supervision is personal supervision in the last 6 months. Regular salary supplement is monthly or daily salary supplement.

## Discussion

This is the first known assessment of the know-do gap in sick child care drawn from a nationally representative sample, and it reveals large gaps in both knowledge and performance of sick child care in Ethiopia. On average providers knew just 39% of assessment, treatment and counseling items they should perform for a child with symptoms of malaria and anemia, and were observed completing only 34% of those items during sick child visits. While doctors have greater knowledge of how to assess the patient than the other cadres, they do not perform these items in practice. The largest gap for all types of providers was in treatment and counseling, which are critical for appropriate disease management.

Provider knowledge was poorly correlated with performance, and approximately one-third of providers actually scored better on performance than on the knowledge test. While knowledge tests have typically been considered the provider’s best practice or upper threshold of performance, these results reveal that the direction of error is not necessarily consistent, making knowledge a poor proxy for estimating clinical quality [13, 20, 21]. Providers scored worse on several vignette items than on the corresponding performance items, particularly in asking about the child’s symptoms. This may be because one of the child’s symptoms, fever, was already specified to the provider in the introduction of the vignette (see S1 Table).

The gap between knowledge and performance can be partially explained by a lack of necessary equipment such as thermometers and scales, the presence of which increase performance specifically for the assessment items of taking the child’s temperature and weighing him. However much of the gap cannot be well explained by the variables observed in this data set. The large differences between doctors and other provider types may be due to differences in pre-service education or the different expectations of the providers in the facilities. Our findings may highlight that physicians perform better on paper tests and that they do not apply this knowledge in practice. This may in part be because physicians in Ethiopia are not formally trained to follow the IMCI protocol, as it is considered a triage tool to be used by lower-level medical personnel [22]. Rather, physicians are expected to rely on clinical judgement, particularly for conditions judged to be less severe. However, clinical judgment may be more accurately considered a complement not a replacement for a detailed assessment, suggesting our findings may reflect deficiencies in medical education that favors learning of pathology over a problem- or competency-based education [23]. Physicians were also more likely to be practicing in hospitals, where they may have a more varied panel of patients under their care beyond sick children. More research is required to understand what other factors may affect the size of the know-do gap, particularly for the in treatment and counseling.

The poor provider performance in sick child care presented in this analysis is consistent with that reported elsewhere in low and middle income countries [4, 8]. Mohanan et al similarly found that the gap was larger for treatment than for patient assessment of sick children in India [9]. Other work demonstrating the know-do gap have identified facility management and intrinsic and extrinsic motivation as key determinants of the know-do gap [10, 12, 24]. These factors were unassociated in our analysis, perhaps because they were poorly defined in the SPA+ data. For example, there is no data collected on the provider’s salaries or salary delays. Interestingly, the lack of relationship between provider caseload and performance has been confirmed elsewhere [24, 25]. The common perception that providers are too overworked to provide adequate care is unfounded, indeed we found facilities had on average only 1.5 sick child patients per provider on the day of the observations. While providers may be have offered other services in addition to sick child care, these findings together suggest that the know-do gap is not due to an overly strenuous workload.

This work has several limitations. First, the vignette presented to providers was designed for a child presenting with fever that has been worsening over time. The providers were observed caring for children with many different symptoms, which may have biased the measure of know-do gap if the provider’s knowledge differed for other conditions. We addressed this by conducting a sensitivity analysis with observations of febrile children, however the symptoms are reported by the caregiver. Second, the lack of provider identifier required us to match based on other variables, and where there was a non-unique match we had to drop part of the sample, which biased our analytic sample toward smaller facilities. While our analytic sample was largely similar to the overall sample of providers providing sick child care, the exclusion of health posts and health extension workers from the knowledge tests prevents us from being able to quantify their know-do gap. Given that health extension workers are a critical part of the primary health care system and provide care to many sick children, they should also receive knowledge tests in future health system assessments. A study of health extension workers found large gaps in how to identify the signs to correctly diagnose sick newborns despite correct diagnosis when told about specific signs, signaling a know-do gap in this cadre as well [26]. Third, the small number of visits observed for each provider (3 on average) limits the heterogeneity of cases, and may be biased upward by the Hawthorn Effect, as provider performance elsewhere has declined after approximately seven observations [13]. Finally, we were unable to verify the accuracy of the provider’s diagnosis in the observations of the clinical care visit, so we are unable to assess the know do gap in diagnostic accuracy. Use of standardized patients rather than observations may address some of these limitations for other types of services [27, 28]. However, standardized patient methods for sick child care typically have caregivers describe their child’s illness, and they would thus be unable to elicit the actions that a health worker would actually perform with the child.

The large deficiencies in the quality of care provided particularly in the treatment and management of sick children suggests that improvement interventions must address not only knowledge, but also provider actions. Common strategies such as in-service training and supervision were unassociated with the know-do gap, signaling that new approaches are needed. These may include higher quality pre-service education, particularly for MDs, which should expose providers to the applicable guidelines and emphasize the common elements of good medical practice across services, including systematic assessment, accurate diagnosis, appropriate treatment and counseling. The Ministry of Health is currently revising basic training guidelines and will place a larger emphasis on developing counseling skills. The Ministry of Health is also introducing a catchment-based mentorship program to link providers at facilities of different levels. It will be important to leverage these mentorships and other supervision efforts to improve provider’s performance. Finally, as the availability of essential inputs and diagnostic kits are key pre-requisites to providing high quality care, a strong supply chain is required to ensure the continuous and sustained availability of such items.

## Supporting information

S1 Tables. Supplementary tables A-E (DOCX)
