# Supplementary material for: The know-do gap in sick child care in Ethiopia
Source: PLoS One. 2018 Dec 12;13(12):e0208898. doi: 10.1371/journal.pone.0208898 (PMC6291134; doi:10.1371/journal.pone.0208898)
Supplement: S1 Tables. Supplementary tables A-E — (DOCX) [file pone.0208898.s001.docx]

**Table A.** Sick child vignette

| Please tell me, what signs, symptoms and other elements of history would you look for in a 4 years old boy presenting with fever that has been worsening over time?  WAIT FOR SPONTANEOUS RESPONSES, THEN PROBE FOR ANY OTHER SIGNS OR SYMPTOMS | Duration of fever  Presence of fever and patter  Shivers or sweats  History of convulsions  History of vomiting  Loss of appetite/changes in eating habits  Diarrhea  Cough  Severity of cough  Type of cough (productive/dry)  Difficulty breathing  Type of medication given  Amount of medication given  Vaccination status to date  Consult a senior staff  Other  Don’t know |
| --- | --- |
| You found out that the boy has had fever for one week, intermittent with shivers and sweats, eats less than usual, vomits sometimes, and has mild dry cough. He was given Panadol to control his fever, one dose two days ago, one yesterday and one this morning; all his vaccinations are up to date. He has had no convulsions, no diarrhea and no difficulty breathing.  What kind of examination would you do?  WAIT FOR SPONTANEOUS RESPONSES, THEN PROBE FOR ANY OTHER ACTIONS | Take temperature  Take weight  Take pulse rate  Take respiratory rate  Check hands (for palmar pallor)  Check tongue  Check eyes (if sunken)  Check eyes (if pale color)  Responsiveness/ General condition  Check skin condition  Check for neck stiffness  Verify if face is puffy  Check for swelling fee  Do abdomen and liver exam  Consult a senior staff  Other  Don’t know |
| Your examination reveals the following:  Temperature: 37.2 degrees Celsius  Weight: 11 Kgs  Pulse rate: 95 per minute, normal respiratory rate  Normal abdomen/liver exam, normal skin.  No neck stiffness. The boy is awake but lethargic. The nail beds are pale, the tongue is also pale. The eyes are not sunken but are pale; the face is not puffy and the feet are not swollen.  Please tell me, what kind of tests would you administer?  WAIT FOR SPONTANEOUS RESPONSES, THEN PROBE FOR ANY OTHER TESTS | Microscopy/blood slide for malaria parasite (BF)  Rapid diagnostic test  Hemoglobin  Full blood count  Consult a senior staff  Other  Don’t know |
| It will take some time to get the results. What would be your preliminary diagnosis?  WAIT FOR SPONTANEOUS RESPONSES, THEN PROBE FOR ANY OTHER DIAGNOSIS | Malaria  Anemia  Malaria with anemia  Severe malaria  Consult a senior staff  Other  Don’t know |
| What treatment would you administer or what would be your action plan?  WAIT FOR SPONTANEOUS RESPONSES, THEN PROBE FOR ANY OTHER TREATMENT | Coartem  Paracetamol/Other fever reducing medicine  Adequate fluid and nutrition  Quinine intravenous infusion  Quinine intramuscular injection  Rectal artesunate (100 MG suppository)  Blood transfusion  Consult a senior staff  Chloroquine  Other  Don’t know |
| What would be your recommendation as part of health education to this patient?  WAIT FOR SPONTANEOUS RESPONSES, THEN PROBE FOR ANY OTHER RECOMMENDATIONS | Adherence to treatment  Prompt return if symptoms worsen  Consult a senior staff  Other  Don’t know |

| **Table B.** Comparison of analytic sample with all providers providing sick child care | | | | | |  |  |
| --- | --- | --- | --- | --- | --- | --- | --- |
|  | Analytic sample of providers  (N = 503) | | All providers providing sick child care (N=3,947) | | | | |
|  | N | percent | N | | percent | |  |
| Facility characteristics |  |  |  | |  | |  |
| Urban | 236 | 47% | 624 | | 47% | |  |
| Basic amenities (mean/sd) | 0.43 | 0.22 | 0.43 | | 0.24 | |  |
| Functional thermometer and scale | 451 | 90% | 939 | | 71% | |  |
| Malaria diagnostics | 366 | 73% | 573 | | 43% | |  |
| Sick child patients per provider (mean/sd) | 1.5 | 2.54 | 0.92 | | 2.28 | |  |
| Management index (mean/sd) | 0.7 | 0.3 | 0.52 | | 0.34 | |  |
| Facility type |  |  |  | |  | |  |
| Hospital | 54 | 11% | 223 | | 17% | |  |
| Health center | 347 | 69% | 298 | | 22% | |  |
| Health post |  |  | 321 | | 24% | |  |
| Higher clinic | 10 | 2% | 70 | | 5% | |  |
| Medium clinic | 33 | 7% | 168 | | 13% | |  |
| Lower clinic | 59 | 12% | 247 | | 19% | |  |
| Provider characteristics |  |  |  | |  | |  |
| Female | 180 | 36% | 2482 | | 63% | |  |
| Years since graduation (mean/sd) | 4.66 | 4.36 | 4.89 | | 3.8 | |  |
| In-service training on sick children | 225 | 45% | 2030 | | 51% | |  |
| Recent supervision | 307 | 61% | 2737 | | 69% | |  |
| Receives regular salary supplement | 408 | 81% | 3094 | | 78% | |  |
| Has written job description | 119 | 24% | 781 | | 20% | |  |
| Aware of opportunities for promotion | 214 | 43% | 1738 | | 44% | |  |
| Provider type |  |  |  | |  | |  |
| MD | 39 | 8% | 105 | | 3% | |  |
| Health officer | 108 | 21% | 281 | | 7% | |  |
| Nurse/Midwife | 356 | 71% | 2044 | | 52% | |  |
| Other provider (Health extension worker,  non-clinical) | | | | 1518 | 38% |  |  |

| **Table C**. Know-do gap for malaria care | | |  |
| --- | --- | --- | --- |
|  | Vignette average | Observed average among febrile children | Gap |
| Assessment items |  |  |  |
| Ask about seizures | 22% | 12% | 10% |
| Ask about vomiting | 36% | 53% | -17% |
| Ask about eating during illness | 36% | 31% | 5% |
| Ask about cough | 59% | 70% | -11% |
| Ask about diarrhea | 36% | 71% | -35% |
| Ask about vaccinations | 11% | 48% | -36% |
| Take temperature | 88% | 78% | 10% |
| Weigh child | 43% | 50% | -7% |
| Check pallor | 15% | 34% | -19% |
| Check for oedema | 8% | 5% | 3% |
| Examine skin | 65% | 31% | 34% |
| Count respirations | 12% | 4% | 8% |
| Examine neck | 17% | 24% | -7% |
| Examine mouth | 17% | 27% | -10% |
| Do a malaria test | 85% | 19% | 67% |
| Assessment average | 37% | 37% | 0% |
| Treatment and counseling items |  |  |  |
| Prescribe artesunate or quinine* | 59% | 38% | 21% |
| Counsel danger signs requiring return | 41% | 20% | 21% |
| Counsel adherence to medication | 58% | 13% | 45% |
| Treatment and counseling average | 53% | 18% | 34% |
| Overall average | 39% | 35% | 5% |
| *Only among children who have been diagnosed with malaria in observed | | |  |

| **Table D.** Association between knowledge and performance   \| Outcome: provider performance \| Beta \| \| 95% CI \| \| \| \| --- \| --- \| --- \| --- \| --- \| --- \| \| Provider knowledge \| \| .145* \| \| [.065,.214] \| \| Urban \| \| -0.025 \| \| [-.051,.003] \| \| Basic amenities \| \| -0.033 \| \| [-.091,.025] \| \| Thermometer and scale \| \| .065* \| \| [1.0e-02,.12] \| \| Malaria diagnostic \| \| 0.0051 \| \| [-.023,.034] \| \| In-service training on sick child care \| \| 0.021 \| \| [-5.5e-04,.043] \| \| Recent supervision \| \| 0.011 \| \| [-.012,.033] \| \| Regular salary supplement \| \| -0.0074 \| \| [-.033,.018] \| \| Provider type \| \| \| \|  \| \| MD \| \| 0.01 \| \| [-.031,.045] \| \| Health Officer \| \| .037** \| \| [.008,.062] \| \| Nurse/Midwife \| \| Ref \| \|  \| \| Constant \| \| .226* \| \| [.159,.283] \| \| N \| \| 503 \| \|  \|   **Table E.** Multivariate predictors of alternate calculations of the know-do gap | | | | |  |  |
| --- | --- | --- | --- | --- | --- | --- | --- | --- | --- | --- | --- | --- | --- | --- | --- | --- | --- | --- | --- | --- | --- | --- | --- | --- | --- | --- | --- | --- | --- | --- | --- | --- | --- | --- | --- | --- | --- | --- | --- | --- | --- | --- | --- | --- | --- | --- | --- | --- | --- | --- | --- | --- | --- | --- | --- | --- | --- | --- | --- | --- | --- | --- | --- | --- | --- | --- | --- | --- | --- | --- | --- | --- | --- | --- | --- | --- | --- | --- | --- | --- | --- | --- |
|  | Fever |  | Sum of discordant items | | |  |
|  | Beta | 95% CI | Beta | 95% CI | | |
| Urban | 0.0 | [-.024,.059] | 0.43 | [-.043,.899] | | |
| Basic amenities | 0.1 | [-.036,.141] | 0.39 | [-.571,1.36] | | |
| Thermometer and scale | -.072* | [-.14,-3.5e-03] | -0.11 | [-.981,.76] | | |
| Malaria diagnostic | 0.0 | [-.049,.039] | 0.06 | [-.436,.559] | | |
| In-service training on sick child care | 0.0 | [-.059,5.9e-03] | 0.01 | [-.377,.394] | | |
| Recent supervision | 0.0 | [-.044,.026] | 0.01 | [-.387,.409] | | |
| Regular salary supplement | 0.0 | [-.02,.069] | 0.17 | [-.385,.72] | | |
| Provider type | |  |  |  | | |
| MD | .11*** | [.058,.163] | .849** | [.264,1.43] | | |
| Health Officer | 0.0 | [-.021,.06] | 0.31 | [-.218,.833] | | |
| Nurse/Midwife | (Reference) |  | (Reference) |  | | |
| Constant | 0.1 | [-.012,.159] | 6.85*** | [5.83,7.86] | | |
| N | 426 |  | 503 |  | | |
